# From Viscount Knutsford

**Published:** 1918-07-20

**Authors:** 

**Affiliations:** Chairman, London Hospital.


### From Viscount Knutsford.

Sir,—I was irritated. It did not seem to me to be playing the game (and in this I observe the Speaker agreed) for a member to make inaccurate statements against this hospital under cover of a question, statements which, by the by, I had given him the opportunity of testing, and as to which there was no one present in the House on behalf of the hospital who knew the answer.

Colonel Maurice in his courteous letter writes that there is no disguising the fact that the nurses are "exploited" to the extent of £6,000 a year. Passing by the somewhat offensive word, this is not so. The last three years show on the receipt side £2,696, £3,428, £2,924. But these amounts only show the sums paid to the hospital, and would disappear altogether if the private nursing was run as a separate organisation outside the hospital. Nothing, for instance, is deducted for rent, rates, and taxes, for a home for 250 nurses, nothing for capital outlay, and so on. It is a matter of bookkeeping, and none too easy at that.

I cannot here discuss with Colonel Maurice the details of our training—you could not grant me the space—but he is wrong in stating that the certificate is not given till the end of the fourth year. It is given when we know that the nurse is fully trained and fit to do responsible nursing work, *i.e.*, at the end of her second year.

It is sufficient for me that we have literally thousands (ten or more) of letters here from doctors and patients expressing their appreciation of our nurses, and that in none of these letters is there any hostile criticism or comment on the training of the nurses, who are, as I said in my former letter, holding to-day the most responsible posts all over the world.

We do not intend to alter our training on the demand of those who cannot get the same results or who do not understand our methods.—Yours faithfully,

July 11. Knutsford, Chairman, London Hospital.

